# The FIB-4 Index Predicts the Development of Liver-Related Events, Extrahepatic Cancers, and Coronary Vascular Disease in Patients with NAFLD

**DOI:** 10.3390/nu15010066

**Published:** 2022-12-23

**Authors:** Yoshihiro Kamada, Kensuke Munekage, Takashi Nakahara, Hideki Fujii, Yoshiyuki Sawai, Yoshinori Doi, Hideyuki Hyogo, Yoshio Sumida, Yasuharu Imai, Eiji Miyoshi, Masafumi Ono

**Affiliations:** 1 Department of Advanced Metabolic Hepatology, Osaka University Graduate School of Medicine, Suita 565-0871, Japan; 2 Department of Gastroenterology and Hepatology, Kochi Medical School, Nankoku 783-8505, Japan; 3 Department of Gastroenterology and Metabolism, Hiroshima University, Hiroshima 734-8553, Japan; 4 Department of Hepatology, Osaka Metropolitan University Graduate School of Medicine, Osaka 545-8585, Japan; 5 Department of Gastroenterology, Ikeda Municipal Hospital, Ikeda 563-8510, Japan; 6 Department of Gastroenterology, Otemae Hospital, Osaka 540-0008, Japan; 7 Hyogo Life Care Clinic Hiroshima, 6-34-1, Enkobashi-cho, Minami-ku, Hiroshima 732-0823, Japan; 8 Department of Gastroenterology and Hepatology, JA Hiroshima General Hospital, Hiroshima 738-8503, Japan; 9 Division of Hepatology and Pancreatology, Department of Internal Medicine, Aichi Medical University, Yazako Karimata, Nagakute 411-1195, Japan; 10 Department of Molecular Biochemistry & Clinical Investigation, Osaka University Graduate School of Medicine, Suita 565-0871, Japan; 11 Division of Innovative Medicine for Hepatobiliary & Pancreatology, Faculty of Medicine, Kagawa University, Takamatsu 761-0793, Japan

**Keywords:** biopsy-confirmed NAFLD, major adverse cardiovascular events (MACEs), biomarker, noninvasive test (NIT)

## Abstract

The prognosis of nonalcoholic fatty liver disease (NAFLD) patients depends on liver-related events (LREs), extrahepatic cancers, and major adverse cardiovascular events (MACEs). The fibrosis-4 (FIB-4) index is one of the most reliable and useful predictors of the degree of liver fibrosis. Recent studies have reported that the FIB-4 index is also useful for predicting LREs and MACEs in NAFLD patients. In the present study, we investigated the prognostic value of the FIB-4 index in NAFLD patients. A total of 506 biopsy-confirmed NAFLD patients from six hepatology centers in Japan from 2002 to 2013 were enrolled in this study. Of these NAFLD patients, 353 were available for more than 100 days of follow-up and did not exhibit events (LREs, extrahepatic cancers, MACEs) at the time of entry. The mean follow-up duration of all the subjects was 2716 ± 1621 days (102–7483 days). New LREs (hepatocellular carcinoma (HCC) (*n* = 8), decompensation (*n* = 11), bleeding varices (*n* = 8)) developed in 18 patients. Twenty-four and twelve patients developed extrahepatic cancers and MACEs, respectively. The median FIB-4 index was 1.255; we divided our cohort into two groups according to this (FIB4 Low, FIB4 Hi). The incidence of HCC tended to be higher in FIB4 Hi (*n* = 7) than in FIB4 Low (*n* = 1). The incidence of LREs was significantly higher in FIB4 Hi (*n* = 17) than in FIB4 Low (*n* = 1). The incidence of extrahepatic cancers was significantly higher in FIB4 Hi (*n* = 20) than in FIB4 Low (*n* = 4); the incidence of MACEs was also significantly higher in FIB4 Hi (*n* = 10) than in FIB4 Low (*n* = 2). The FIB-4 index is a useful biomarker for predicting not only LREs but also extrahepatic cancers and MACEs.

## 1. Introduction

Nonalcoholic fatty liver disease (NAFLD) is the most common cause of chronic liver disease and a growing medical problem worldwide [1]. NAFLD has a wide range of histological changes, from nonalcoholic fatty liver (NAFL) to nonalcoholic steatohepatitis (NASH). Assessment of the liver fibrosis degree during the NAFLD progression is critical to predict disease severity and make therapeutic strategies [2,3]. Recent studies have reported that liver fibrosis is independently associated with long-term prognosis in patients with NAFLD [4]. Liver biopsy is still the gold standard for evaluating liver fibrosis [5,6], but it comes with significant limitations, including pain, risk of serious complications, sampling errors [7], cost [8], and patients unwilling to undergo invasive testing. Therefore, useful and reliable noninvasive tests (NIT) are needed to assess the degree of disease progression and to predict the outcome of NAFLD patients in advance.

The prognosis of patients with NAFLD depends on liver-related events (LREs) and diseases of extrahepatic organs, such as extrahepatic cancer and cardiovascular disease (CVD) [4,9]. These findings are based on studies conducted in the United States and Europe that primarily evaluated Caucasian patients with NAFLD and in which CVD was the leading cause of death and event occurrence. Simon et al. studied the prognosis of patients with NAFLD over a median of 13.6 years and found a major adverse cardiovascular event (MACE) rate of 24.3/1000 person-years (PY). This incidence was higher than in the control group (8.3/1000 PY) [9].

We recently conducted a multicenter, registry-based, retrospective cohort study of patients diagnosed with NAFLD on biopsy (CLIONE in Asia, *n* = 1398) [10]. A median of 4.6 years, 77 (5.5%), 71 (5.1%), and 66 (4.7%) patients developed LREs, MACEs, and extrahepatic cancers, respectively. The incidence of LREs, MACEs, and extrahepatic cancers was 8.7/1000 PY, 8.0/1000 PY, and 7.4/1000 PY, respectively. A Korean study followed more than 25,000 NAFLD patients for 7.5 years and found that patients with NAFLD were more likely to develop three cancers (hepatocellular carcinoma (HCC), colorectal cancer in male patients, and breast cancer in female patients) [11]. In addition, a recent systematic review has shown that NAFLD is associated with an increased risk of CVD and extrahepatic cancer [12]. In a recent meta-analysis, NAFLD is also demonstrated as an independent risk factor for myocardial infarction, heart failure, atrial fibrillation, and ischemic stroke [13]. In 2022, the American Heart Association (AHA) demonstrated that NAFLD is an independent risk factor for CVD in a scientific statement [14].

Among various scoring systems for predicting the degree of liver fibrosis in NAFLD patients, the fibrosis-4 (FIB-4) index is one of the most reliable and useful in clinical usage [15,16]. Recent studies from Western countries have reported that the FIB-4 index is useful for predicting the liver-related events (LREs) of NAFLD patients [17,18]. In addition, recent studies have reported that the FIB-4 index is also useful for predicting CVD events [19]. In type 2 diabetes mellitus patients, the FIB-4 index can stratify the risk of developing CVD events [20]. FIB-4 index is well-known as a convenient scoring system and is expected as one of the most reliable and useful NITs [21].

Considering both these findings, the FIB-4 index should be useful for predicting not only LREs but also other organ diseases (CVD, extrahepatic cancers). In the present study, the prognosis of 506 NAFLD patients since their FIB-4 index was measured by liver biopsy was followed up. In this study, we examined the prognostic value of the FIB-4 index in patients with NAFLD to investigate the ability of the FIB-4 index as an NIT.

## 2. Patients and Methods

### 2.1. Ethical Committee Approval

The protocol and informed consent were approved as a multicenter study by the Institutional Review Board of Osaka University Hospital, Kochi Medical School Hospital, Osaka Metropolitan University Hospital, Hiroshima University Hospital, Ikeda Municipal Hospital, and Otemae Hospital. Written informed consent was obtained from all subjects at the time of liver biopsy or enrollment at each site. This study was conducted in accordance with the Declaration of Helsinki.

### 2.2. Biopsy-Confirmed NAFLD Patients and Histological Evaluation

**Figure 1:**
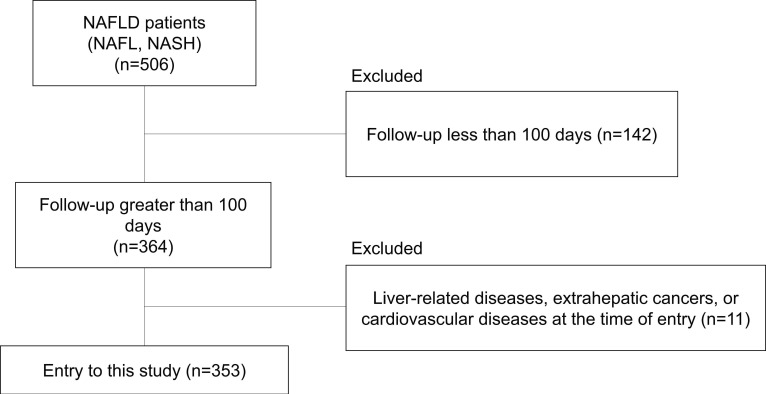
Flow diagram of patient enrollment throughout the study.

A total of 506 patients with biopsy-confirmed NAFLD were enrolled in the study from 2002 to 2013 at the following six liver disease centers in Japan: Osaka University Hospital, Kochi Medical School Hospital, Osaka Metropolitan University Hospital, Hiroshima University Hospital, Ikeda Municipal Hospital, and Otemae Hospital. The study included 364 patients with NAFLD who could be followed up for more than 100 days at each hospital, who had a second or subsequent outpatient visit, and who agreed to participate in the present study (Figure 1). Of these 364 patients, 11 were excluded due to liver-related diseases, extrahepatic cancer, or CVD at entry, and 353 were enrolled in this study.

All patients with biopsy-confirmed NAFLD underwent percutaneous liver needle biopsy. Following standard procedures, biopsied liver specimens were embedded in paraffin blocks and stained with hematoxylin, eosin, and Masson’s trichrome stain. All biopsy specimens were centrally evaluated by two experienced liver pathologists (Y.K. and H.F.) blinded to clinical data. Appropriate specimens were defined as >1.5 cm in length or >6 portal veins.

NASH was identified according to Matteoni’s classification [22]. Patients with NAFLD with balloon hepatocytes (Matteoni type 3) and NAFLD with liver fibrosis (Matteoni type 4) were placed in the NASH cohort. Patients whose liver biopsy specimens showed simple lipidosis or lipidosis with nonspecific inflammation were placed in the NAFL cohort. Samples were also investigated and quantified according to the NAFLD activity scoring (NAS) system [23]. Stethosis (0–3), lobular inflammation (0–2), and hepatocyte ballooning (0–2) were quantified. Individual parameters of fibrosis were scored independently according to the NASH Clinical Research Network scoring system [23]. Exclusion criteria for this study were liver-related disease at entry (HCC, compensated cirrhosis, bleeding gastroesophageal varices), other liver disease, liver damage due to substance abuse, and a history of alcohol abuse (defined as a daily alcohol intake of 20 g or more).

### 2.3. Definition of LREs (HCC, Decompensation, and Bleeding Gastroesophageal varices) and MACEs

In this study, all clinical events were collected and defined using data from electronic medical records. LREs (HCC, decompensation, and bleeding gastroesophageal varices) were defined as follows. HCC was confirmed by showing typical features on (1) histology or (2) at least one dynamic examination (three-phase computed tomography (CT) or magnetic resonance imaging (MRI)) according to the guidelines of the Japanese Society of Hepatology (JSH) [24]. Decompensation was defined as decompensated liver cirrhosis. The date of initial admission due to ascites or hepatic encephalopathy was recorded. Ascites was confirmed by (1) the detection of ascites by aspiration and (2) radiological examination (ultrasonography, CT, MRI). Bleeding gastroesophageal varices were recorded as diagnosed on the first admission for variceal treatment. Coronary events were defined as hospitalization for stable angina, unstable angina, myocardial infarction, or sudden cardiac arrest. In this study, MACEs included coronary events, heart failure, and stroke.

### 2.4. Anthropometry and Laboratory Measurements

Anthropometric variables (height and weight) were measured in the standing position, and the body mass index (BMI) was calculated as weight (kg)/height squared (m^2^). Serum biochemical variables (aspartate aminotransferase (AST), alanine aminotransferase (ALT), γ-glutamyl transferase (GGT), alkaline phosphatase (ALP), total cholesterol (T-Chol), triglycerides (TG), high-density lipoprotein cholesterol (HDL-C), fasting blood sugar (FBS), immunoreactive insulin (IRI), albumin (Alb), ferritin, hyaluronic acid, and platelet count) were measured using conventional automated analyzers. The FIB-4 index was calculated for each of the subjects as previously reported (age × AST (U/L)/platelet count (×10^9^/L)/√ALT (U/L)) [15,16].

### 2.5. Statistical Analysis

Statistical analysis was performed using JMP Pro 16.2 software (SAS Institute Inc., Cary, NC, USA). Variables were expressed as mean ± standard deviation. Clinical outcomes were presented as Kaplan–Meier curves and compared by log-rank test. The diagnostic performance of the markers was evaluated by analysis of receiver operating characteristic (ROC) curves. The measurement probabilities of true positive (sensitivity) and true negative (specificity) were determined for the selected cutoff values, and the area under the ROC curve (AUC) was calculated for each indicator. The Youden index was used to identify the optimal cutoff value. Differences were considered statistically significant at *p* < 0.05.

## 3. Results

### 3.1. Characteristics of the Study Subjects

Of the 506 patients with NAFLD, 353 were monitored for more than 100 days, had a second or subsequent outpatient visit, and agreed to participate in the present study (Figure 1). The characteristics of the study subjects are shown in Table 1. The median value of the FIB-4 index value was 1.255, and we divided our cohort into two groups using the median FIB-4 index value (FIB-4 index low group (FIB4 Low), FIB-4 index high group (FIB4 Hi)). In our cohorts, age, AST, ALT, AST/ALT ratio, ALP, ferritin, and hyaluronic acid were significantly higher in the FIB4 Hi group than in the FIB4 Low group. The BMI, albumin, and platelet count were significantly lower in the FIB4 Hi group than in the FIB4 Low group. The FIB4 Hi group had a higher proportion of advanced liver fibrosis (F3-4) patients than the FIB4 Low group.

### 3.2. Follow-Up Evaluation

The mean follow-up period for all subjects was 2716 ± 1621 days (102–7483 days, or about 7.4 years). This cohort corresponds to 2626.3 PY for all subjects, 1431.9 PY for FIB4 Hi patients, and 1194.3 PY for FIB4 Low patients. Ten patients died. One liver-related death was cholangiocellular carcinoma (CCC), and the other nine patients died from a variety of causes (lung cancer, breast cancer, stomach cancer, pneumonia, heart failure, subarachnoid hemorrhage, congestive heart failure, pancreatic cancer, and acute myeloid leukemia).

Table 2 shows the main complications and their incidence. In our cohort, 18 (5.1%) had new liver-related events, 12 (3.4%) had new MACEs, and 24 (6.8%) had new cancer in extrahepatic organs. The incidence of these events is shown in Table 2. The incidence of death was similar between the two groups. The incidence of HCC tended to be higher in FIB4 Hi than in FIB4 Low (0.84 vs. 4.89/1000 PY, *p* = 0.07). The rates of decompensation, varices, and liver-related events were significantly higher in FIB4 Hi than in FIB4 Low (0.00 vs. 7.68/1000 PY, 0.00 vs. 5.59/1000 PY, and 0.84 vs. 11.87/1000 PY, respectively). The rates of MACEs and extrahepatic cancer were also significantly higher in FIB 4 Hi than FIB4 Low (1.67 vs. 7.68/1000 PY and 6.70 vs. 16.76/1000 PY, respectively). Among extrahepatic cancers, the incidence of colorectal cancer tended to be higher in FIB 4 Hi than in FIB 4 Low (0.00 vs. 3.49/1000 PY, *p* = 0.06).

### 3.3. Incidence of LREs

**Figure 2:**
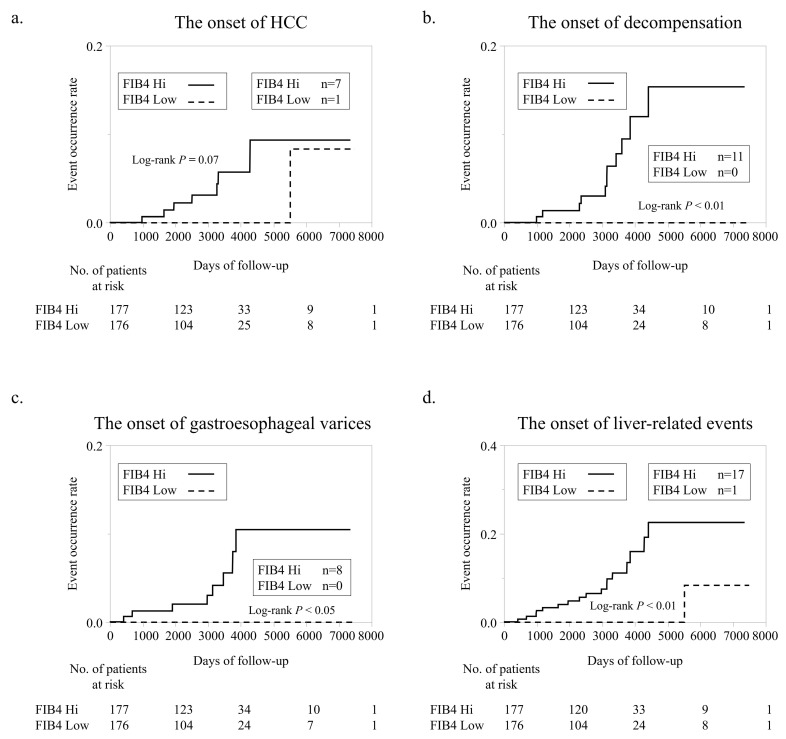
Liver-related event development according to FIB-4 index. (**a**). Comparison of new HCC development according to FIB-4 index. (**b**). Comparison of new decompensation development according to FIB-4 index. (**c**). Comparison of new bleeding gastroesophageal varices development according to FIB-4 index. (**d**). Comparison of LRE development according to FIB-4 index.

The cumulative probability of LREs (HCC, decompensation, varices) between the FIB4 Low and FIB4 Hi groups obtained by Kaplan–Meier analysis is shown in Figure 2. The incidence of HCC tended to be higher in the FIB4 Hi group (*n* = 7) than in the FIB4 Low group (*n* = 1) (Figure 2a). The incidence of decompensation was significantly higher in FIB4 Hi (*n* = 7) than in FIB4 Low (*n* = 1) (Figure 2a). The incidence of hemorrhagic varices was significantly higher in FIB4 Hi (*n* = 8) than in FIB4 Low (*n* = 0) (Figure 2c); the incidence of LREs was significantly higher in FIB4 Hi (*n* = 17) than in FIB4 Low (*n* = 1) (Figure 2d).

**Figure 3:**
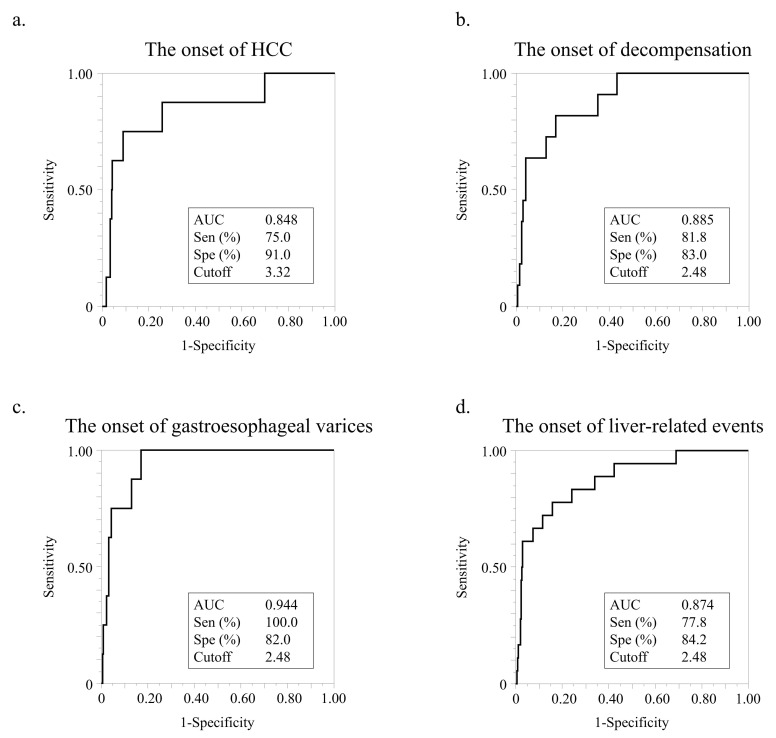
ROC analysis of FIB-4 index for liver-related events. (**a**) ROC analysis of FIB-4 index for HCC development. (**b**) ROC analysis of FIB-4 index levels for decompensation development. (**c**) ROC analysis of FIB-4 index for bleeding gastroesophageal varices development. (**d**). ROC analysis of FIB-4 index for LRE development.

Using ROC analyses, we set cutoff values for the FIB-4 index value for HCC, decompensation, varices, and LREs (Figure 3). The cutoff value for HCC occurrence was 3.32, and the AUC, sensitivity, and specificity of this cutoff value were 0.848, 75.0%, and 91.0%, respectively (Figure 3a). The cutoff value for the occurrence of decompensation was 2.48, and the AUC, sensitivity, and specificity of this cutoff value were 0.885, 81.8, and 83.0%, respectively (Figure 3b). The cutoff value for variceal development was also 2.48, and the AUC, sensitivity, and specificity of this cutoff value were 0.944, 100.0%, and 82.0%, respectively (Figure 3c). The cutoff value for LREs was also 2.48, and the AUC, sensitivity, and specificity of this cutoff value were 0.874, 77.8%, and 84.2%, respectively (Figure 3d).

The occurrence of LREs (HCC, decompensation, varices, and total LREs) was compared with the FIB-4 index and degree of liver fibrosis (Table 3). No association was found between the development of HCC and the FIB-4 index in either early (F0–2) or advanced (F3–4) stages. In advanced-stage patients, the incidence of decompensation was significantly higher in the FIB4 Hi group than in the FIB4 Low group. The FIB4 Hi group also tended to be higher in the development of varices. The total LRE development in early-stage patients was significantly higher in the FIB4 Hi group than in the FIB4 Low group.

### 3.4. Incidence of Extrahepatic Cancers

**Figure 4:**
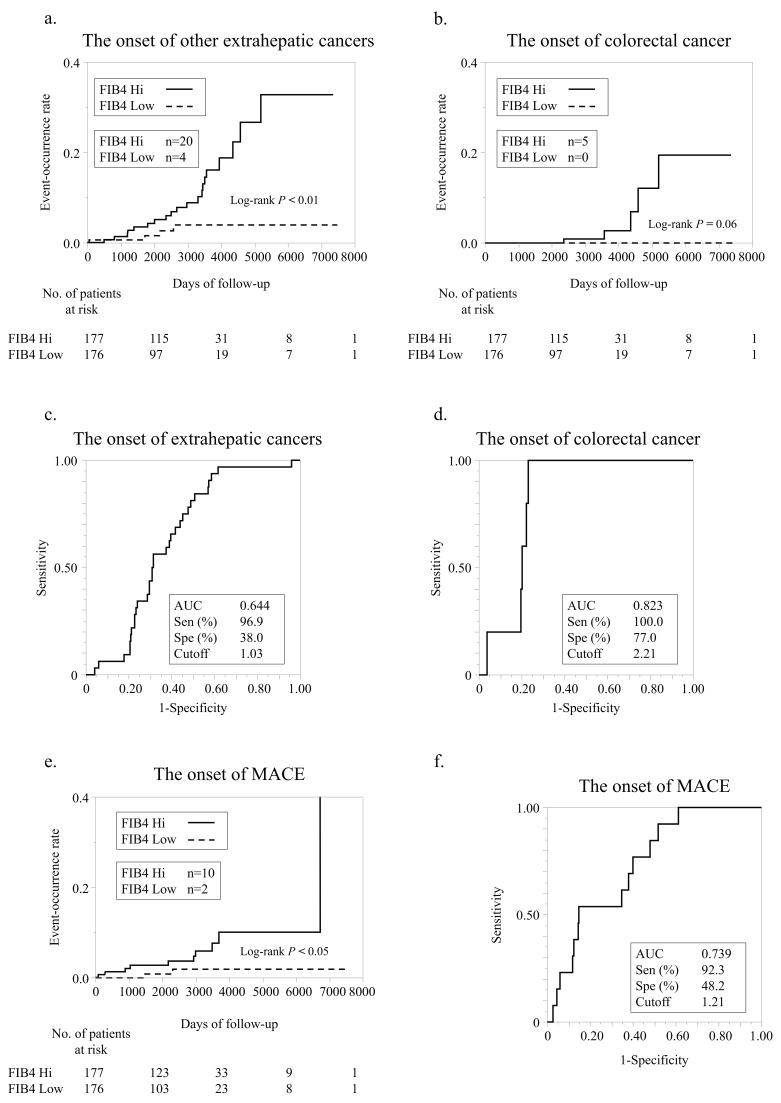
Extrahepatic cancer development according to FIB-4 index. (**a**) Comparison of new extrahepatic cancer development according to FIB-4 index. (**b**) Comparison of new colorectal cancer development according to FIB-4 index. (**c**) ROC analysis of FIB-4 index for extrahepatic cancer development. (**d**) ROC analysis of FIB-4 index for colorectal cancer development. (**e**) Comparison of new MACE development according to FIB-4 index. (**f**) ROC analysis of FIB-4 index for MACE development.

In our cohort, 24 patients developed new extrahepatic cancers. The FIB4 Hi group demonstrated a significantly higher incidence of new extrahepatic cancers (*n* = 20) than the FIB4 Low group (*n* = 4) (Figure 4a). Details of extrahepatic cancers are given in Table S1. Among the various extrahepatic cancers, NAFLD is considered a risk factor for colorectal cancer [11,25]. The incidence of new colorectal cancer was higher in FIB4 Hi (*n* = 5) than in FIB4 Low (*n* = 0). In the FIB4 Low group, no new colorectal cancer patients were observed (Figure 4b). Using ROC analyses, we set cutoff values for the FIB-4 index value for extrahepatic and colorectal cancers (Figure 4c,d). The cutoff value for the incidence of extrahepatic cancer was 1.03, and the AUC, sensitivity, and specificity for this cutoff value were 0.644, 96.9%, and 38.0%, respectively (Figure 4c). The cutoff value for colorectal cancer incidence was 2.21, and the AUC, sensitivity, and specificity for this cutoff value were 0.823, 100.0%, and 77.0%, respectively (Figure 4d).

We compared the development of extrahepatic cancers and colorectal cancer using the FIB-4 index and the liver fibrosis degree (Table 3). There was no association between FIB-4 index and the development of colorectal cancer in both early and advanced stages. The incidence of extrahepatic carcinoma in early-stage patients was significantly higher in the FIB4 Hi group than in the FIB4 Low group.

### 3.5. Incidence of MACEs

In our cohort, 12 patients developed new MACEs. The FIB4 Hi group demonstrated a significantly higher incidence of new MACEs (*n* = 10) than the FIB4 Low group (*n* = 2) (Figure 4e). Using ROC analysis, we established a cutoff value for the FIB-4 index value for MACEs (Figure 4f). The cutoff value for extrahepatic carcinogenesis was 1.21, and the AUC, sensitivity, and specificity of this cutoff value were 0.739, 92.3%, and 48.2%, respectively.

The FIB-4 index and degree of liver fibrosis were used to compare the development of MACEs (Table 3). In advanced-stage patients, there was no association between MACE development and FIB-4 index. However, the incidence of MACEs in early-stage patients was significantly higher in the FIB4 Hi group than in the FIB4 Low group.

## 4. Discussion

In the present study, the FIB-4 index value predicted not only future LREs (HCC, decompensation, bleeding gastroesophageal varices) but also future extrahepatic cancers and MACEs. The FIB-4 index was especially useful for predicting decompensation and bleeding varices. In our study, none of the NAFLD patients with a low FIB-4 index (FIB4 Low) developed decompensation and bleeding varices. Additionally, the FIB-4 index predicted future extrahepatic cancer occurrence and MACEs. None of the FIB4 Low patients developed colorectal cancer.

LREs, extrahepatic cancers, and MACEs are important events for the prognosis of NAFLD patients [4,11,26]. Our recent multicenter cohort study (CLIONE in Asia, *n* = 1398) demonstrated that for a median observation period of 4.6 years, 77, 66, and 71 patients developed LREs, extrahepatic cancers, and MACEs, respectively. In the present study, we set FIB-4 index cutoff values for HCC, decompensation, varices, extrahepatic cancer, and MACE development using ROC analyses. The cutoff values for LREs were relatively high (HCC 3.32, decompensation 2.48, varices 2.48), and the cutoff values for extrahepatic cancers and MACEs were relatively low (extrahepatic cancers 1.03, MACEs 1.21). The FIB-4 index was especially useful for predicting extrahepatic cancers and MACEs in early-stage liver fibrosis (Table 3). Even in early-stage liver fibrosis NAFLD patients, patients with a high FIB-4 index benefit from vigilant monitoring for the development of extrahepatic cancers and MACEs.

NAFLD is becoming the leading cause of HCC worldwide. In a study based in Korea, Kim et al. investigated 25,947 NAFLD patients with a median follow-up of 7.5 years [11]. They demonstrated that NAFLD was associated with developing HCC (hazard ratio (HR) 16.73). They also found that male and female NAFLD patients showed a higher association with the development of colorectal cancer (HR 2.01) and breast cancer (HR 1.92), respectively.

In the CLIONE study based in Asia, the leading cause of death was extrahepatic cancers [10]. Thus, developing an enclosure method for high-risk extrahepatic cancers in NAFLD patients is important, especially in Asian countries. In this study, 24 (6.8%) patients developed extrahepatic cancers. Two-thirds (16/24) of the extrahepatic cancer patients were in the early stage of liver fibrosis. In early-stage NAFLD patients, the FIB4 Hi patients demonstrated a significantly higher incidence of extrahepatic cancers (Table 3). Among extrahepatic cancers, colorectal cancer is a metabolic syndrome-related cancer [25,27], and NAFLD is closely associated with the development of colorectal cancer [11]. Numerous epidemiological studies have shown that NAFLD is significantly associated with the risk of colorectal adenocarcinoma and cancer [28]. The present study demonstrates that a high FIB-4 index value is a useful biomarker for predicting the development of colorectal cancer in patients with NAFLD. Furthermore, no colorectal cancer occurred in the FIB4 Low patients during the observation period. The incidence of colorectal cancer was significantly higher in advanced-stage patients than in early-stage patients in the FIB4 Hi group (*p* < 0.05).

In this study, the MACE incidence in NAFLD patients was 4.57/1000 PY. This incidence is very low compared with the incidence in Caucasian NAFLD patients and comparable to the data from the Caucasian general population [9]. In Asian countries, CVD-related mortality rates were lower than in Western countries [4,9,29,30]. Asian NAFLD patients would thus have a different prognosis from Caucasian NAFLD patients. In our present study, 12 NAFLD patients developed MACEs during the observation period, and the FIB4 Hi group developed MACEs significantly more frequently than the FIB4 Low group. Ten of twelve (83.3%) NAFLD patients who developed MACEs were early-stage fibrosis patients, and the FIB4 Hi group demonstrated significantly higher MACE incidence than the FIB4 Low group in early-stage patients. These findings demonstrated that extrahepatic events (cancers, MACEs) occur in early-stage NAFLD patients with higher FIB-4 index values. Even in the early stage, clinicians should be aware of the potential for other organ events in NAFLD patients with higher FIB-4 index values.

Our study has several limitations. The first is the relatively short follow-up period for monitoring the survival of NAFLD patients. Second, the relatively small number of patients may explain why statistical analysis of the follow-up data did not show significant differences. Third, we did not measure the patatin-like phospholipase domain-containing protein 3 (*PNPLA3*) gene polymorphism, which is more common in Asians than in Westerners [31]. This genetic polymorphism has a homozygous mutation in about 20% of the general Japanese population [32] and is associated with both the development and progression of NAFLD [33,34].

## 5. Conclusions

In conclusion, FIB-4 index values are a useful and reliable NIT to predict not only LREs but also extrahepatic events (cancers, MACEs). In future research, longer and larger follow-up studies are needed to examine the predictive ability of the FIB-4 index for complications associated with NAFLD.

## Figures and Tables

**Table 1:** Liver biopsy data of study subjects.

Variable	FIB4 Low (<1.255)	FIB4 Hi (≥1.255)	*p* Value *
Number	176	177	
Age (y)	43.5 ± 11.7	60.6 ± 8.5	<0.0001
Sex (F/M)	91/85	93/84	0.875
BMI (kg/m^2^)	28.1 ± 5.0	27.0 ± 4.6	0.041
AST (U/L)	44.1 ± 33.8	72.2 ± 44.1	<0.0001
ALT (U/L)	86.1 ± 73.2	96.7 ± 65.5	0.0037
AST/ALT ratio	0.580 ± 0.174	0.802 ± 0.242	<0.0001
GGT (U/L)	92.3 ± 99.8	79.1 ± 69.2	0.390
ALP (U/L)	238.5 ± 102.0	281.3 ± 112.4	<0.0001
T-Chol (mg/dL)	205.1 ± 35.9	205.4 ± 38.6	0.734
TG (mg/dL)	135.5 ± 75.0	133.7 ± 54.2	0.504
HDL-C (mg/dL)	52.1 ± 12.5	48.8 ± 11.4	0.190
FBS (mg/dL)	107.0 ± 17.9	104.4 ± 20.0	0.192
IRI (mU/mL)	14.4 ± 9.2	11.5 ± 5.8	0.281
Albumin (g/dL)	4.54 ± 0.39	4.36 ± 0.44	<0.0001
Ferritin (ng/mL)	231.1 ± 214.1	312.5 ± 324.1	0.023
Hyaluronic acid (ng/mL)	26.6 ± 15.9	130.9 ± 244.0	<0.0001
Platelet count (×10^4^/μL)	26.2 ± 20.7	18.9 ± 5.2	<0.0001
FIB4-index	0.82 ± 0.27	2.52 ± 1.24	<0.0001
Stage (0/1/2/3/4)	45/57/28/13/0	19/37/62/53/5	<0.0001

* FIB4 Low vs. Hi patients. The median value of the FIB-4 index value was 1.255, and we divided our cohort into two groups using the median FIB-4 index value.

**Table 2:** Incidence rate per 1000 person-years and number of events.

	FIB4 Low	FIB4 Hi	Odds Ratio (95% CI)	*p* Value *
Death (*n*)	1.67 (2)	5.59 (8)	4.12 (0.86–19.67)	0.131
HCC (*n*)	0.84 (1)	4.89 (7)	7.21 (0.88–59.19)	0.071
Decompensation (*n*)	0.00 (0)	7.68 (11)	Incalculable **	0.0049
Varices(*n*)	0.00 (0)	5.59 (8)	Incalculable **	0.015
Liver-related disease (*n*)	0.84 (1)	11.87 (17)	18.59 (2.44–141.31)	0.0009
MACEs (*n*)	1.67 (2)	7.68 (10)	5.76 (1.26–26.40)	0.041
Extrahepatic cancers (*n*)	6.70 (4)	16.76 (20)	3.29 (1.44–7.55)	0.0095
Colorectal cancer (*n*)	0.00 (0)	3.49 (5)	Incalculable **	0.059

The median FIB-4 index was 1.255; we divided our cohort into two groups according to this [FIB4 Low (*n* = 176), FIB4 Hi (*n* = 177)]. * Log-rank test. ** Odds ratio and 95% CI (confidence interval) were incalculable due to the lack of control case events. HCC: confirmed by histology or CT/MRI, Varix: patients who need hospitalization (rupture and/or preventive therapy), MACEs: includes coronary artery events, heart failure, and stroke.

**Table 3:** Comparison of the number of each event by FIB-4 index and degree of liver fibrosis.

	FIB4 Low	FIB4 Hi	*p* Value *
HCC			
early stage (F0–2)	0/163	0/119	Incalculable **
advanced stage (F3–4)	1/13	7/58	0.64
Decompensation			
early stage (F0–2)	0/163	2/119	0.063
advanced stage (F3–4)	0/13	9/58	0.048
Varices			
early stage (F0–2)	0/163	1/119	0.19
advanced stage (F3–4)	0/13	7/58	0.083
LREs			
early stage (F0–2)	0/163	3/119	0.022
advanced stage (F3–4)	1/13	14/58	0.15
Extrahepatic cancers			
early stage (F0–2)	3/163	13/119	0.0057
advanced stage (F3–4)	1/13	7/58	0.53
Colorectal cancer			
early stage (F0–2)	0/163	1/119	0.19
advanced stage (F3–4)	0/13	4/58	0.20
MACEs			
early stage (F0–2)	2/163	8/119	0.013
advanced stage (F3–4)	0/13	3/58	0.26

* FIB4 Low vs. Hi patients. ** *p* values were incalculable due to the lack of events.

## Data Availability

Data is unavailable due to privacy or ethical restrictions.

## Supplementary Materials

The following supporting information can be downloaded at: https://www.mdpi.com/article/10.3390/nu15010066/s1, Table S1: Details of extrahepatic cancers in our cohort.
