# Supplementary material for: The FIB-4 Index Predicts the Development of Liver-Related Events, Extrahepatic Cancers, and Coronary Vascular Disease in Patients with NAFLD
Source: Nutrients. 2022 Dec 23;15(1):66. doi: 10.3390/nu15010066 (PMC9824239; doi:10.3390/nu15010066)
Supplement: Supplementary file 1 [file nutrients-15-00066-s001.zip › nutrients-2084303-supplementary.pdf]

Table S1. Details of extrahepatic cancers in our cohort

	GIST	Merkel cell carcinoma	Breast cancer	Colorectal cancer	Uterine cancer	Leukemia	Cholangiocarcinoma	Lung cancer	Stomach cancer	GB cancer	Pleural mesothelioma	Pancreas IPMA	Pancreas cancer	Total
**FIB4 Low**	0	0	0	0	1	1	1	0	1	0	0	0	0	4
**FIB4 Hi**	1	1	2	5	1	0	0	1	5	1	1	1	1	20
**Total**	1	1	2	5	2	1	1	1	6	1	1	1	1	24

GIST, Gastrointestinal Stromal Tumor; GB, gallbladder; IPMA, intraductal papillary mucinous adenoma.
